# Caries arrest and lesion appearance using two different silver fluoride therapies on primary teeth with and without potassium iodide: 12‐month results

**DOI:** 10.1002/cre2.367

**Published:** 2020-12-28

**Authors:** Bathsheba Turton, Rithvitou Horn, Callum Durward

**Affiliations:** ^1^ Faculty of Dentistry University of Puthisastra Phnom Penh Cambodia

**Keywords:** arrest of caries, lesion appearance, potassium iodide, silver diamine fluoride

## Abstract

**Background:**

Globally, has been an increase in the use of silver fluoride products to arrest carious lesions and a variety of products are available.

**Objectives:**

To examine differences in caries arrest and lesion colour of primary tooth carious lesions.

**Material and Methods:**

A four‐armed, parallel‐design cluster‐randomised controlled trial which investigated four protocols for caries arrest at 6m and 12m. Children in Group 1 and Group 2 received Rivastar Silver Diammine Fluoride (SDF), and children in Group 3 and Group 4 received a stabilised aqueous silver fluoride solution (AgF). Children in Group 2 and Group 4 received an additional application of KI immediately after the fluoride. Differences in caries arrest and lesion appearance were examined at 6m and 12m using two level logistic regression modelling.

**Results:**

The arrest rate varied by group membership; group 1 and group 3 had higher arrest rates (77.3% and 75.3% respectively) than group 2 and group 4 (65.4% and 51.2% respectively). The use of KI was also associated with lower odds of arrest (12m OR 0.25; CI 0.19, 0.34) and higher odds of avoiding black discolouration (12m OR 6.08; 2.36, 15.67).

**Conclusions:**

Globally, has been an increase in the use of silver fluoride products to arrest carious lesions and a variety of products are available. This study demonstrated that both AgF and SDF can effectively arrest carious lesions on primary teeth. The use of KI is associated with poorer caries control but better aesthetic outcomes.

## 1 INTRODUCTION

Over the last 10‐years there has been a resurgence in the use of silver fluoride products to manage carious lesions, especially within the primary dentition. The caries arrest activity of silver fluoride products occurs through two primary mechanisms: (1) the fluoride enhances mineral formation and hardening of the tooth, and (2) there is an inhibition of the enzymes that break down tooth structure (matrix metalloproteinase inhibition). Systematic reviews suggest that clinicians could expect between 50% and 70% arrest rates after two applications of silver fluoride in preschool or primary school‐aged children, respectively (Seifo et al., 2019). Arrest rates with two applications is higher. With this evidence, the use of silver fluoride products has been recommended as a key technique in the nonrestorative management of carious lesions in primary teeth (Duangthip et al., 2015; Slayton et al., 2018; Urquhart et al., 2019).

Among higher‐income countries nonrestorative or minimally invasive care is seen as a way of reducing the need for dental rehabilitation under general anesthetic (GA) and as a way of helping children participate in dental treatment (Arrow, 2015). Within low or middle‐income settings, it has been used as a way to manage dental caries in primary teeth in a cost‐effective, appropriate and acceptable way (Chu & Lo, 2008; Turton, Patel et al., 2020; Yee et al., 2009). In Cambodia, silver fluoride solutions are increasingly being used to manage carious lesions on primary teeth; limited resources for restorative care are focused on permanent teeth where long term preservation and function are prioritized. This strategy means that more children can receive care and a higher proportion of carious lesions can be managed within a high caries population (Turton, Patel, et al., 2020).

While silver fluoride treatments are becoming more broadly used, they are not without challenges, particularly around caregiver acceptance. Reports suggest that a large proportion of parents or caregivers are reconciled to acceptance of the black staining that accompanies this approach, given the relative ease of the procedure compared with conventional restorative approaches and the fact that they may be able to avoid submitting their child to rehabilitative treatment under GA (Crystal et al., 2017). Since black staining of the silver fluoride‐treated lesions is the major drawback, in recent years several researchers and companies have sought to address this issue. There are two published techniques for improving the aesthetics of topical silver applications; the first involves the use of nanosilver particles (Tirupathi et al., 2019), and the second involves the use of potassium iodide to remove excess ionized silver (Knight et al., 2006; Zhao et al., 2017).

Along with those options for modifying the color of the lesions, there are also a growing number of silver products available on the market. While it is clear that those products with less than 30% silver ion concentration are less likely to be therapeutic, other questions remain around which base solutions might work best (Fung et al., 2016). The silver and the fluoride components are normally carried in an alkaline solution and a number of different carriers have been used. The high pH amine‐based solutions are more likely to irritate the pulp and so it would be preferable to use solutions which are less alkaline. The other issue concerning products with an ammonia base is that they can become less stable over time, especially when transported in smaller quantities. For that reason, this investigation sought to examine caries arrest and lesion color outcomes using two different silver fluoride solutions (stabilized aqueous silver fluoride [AgF] and silver diamine fluoride [SDF]), with and without KI.

## 2 METHODS

This was a four‐armed, parallel‐design cluster‐randomized controlled trial that followed the CONSORT conventions for reporting of clinical trials. The protocol can be accessed through the International Standard Randomised Controlled Trials Number Registry (ISRCTN87596444). The study recruited children from four schools who were scheduled to join the Healthy Kids Cambodia (HKC) Programme in the 2020–2021 academic year. The investigation facilitated early access to the program for children at each school and as such the study did not employ a nontreatment group but rather a noninferiority design. The four schools involved were Ang Sleng and Monirangsey primary schools in Takeo province, and Neareay and Taten primary schools in Kampot province, Cambodia. Children were randomized at school level for allocation into the four treatment groups. Randomization at school level was performed by the research assistant (RH) using the “pull out of a hat” method. Those children who did not meet the inclusion criteria received routine HKC treatment and management; in addition, those who were identified as needing urgent care were referred for more advanced management.

### 2.1 Ethical considerations

The HKC Programme had an existing ethical approval from the National Ethics Committee for Health Research to formerly observe and monitor the treatment provided including placement of SDF (Review number: 209NECHR). The present study involved a clinical protocol that varied from the standard HKC treatment regime, moving away from an observational study design toward an experimental design. For that reason, an additional ethical review and approval was required. The experimental protocol was reviewed and approved by the internal Research Committee of the University of Punthisastra and by the National Ethics Committee for Health Research, Ministry of Health, Cambodia.

Children in the study received two applications of silver fluoride (at baseline and at the 6‐month follow‐up) and were examined at the same time points, as well as at 12 months. In addition, the schools were supported in conducting a daily handwashing and tooth brushing routine, with the HKC project providing training and materials (tooth brushes, toothpaste, soap, and toothbrush holders).

The consent process involved multiple steps due to the fact that some parents had limited literacy. The first step involved group level consent and distribution of information through the school support committee; the second step involved a written consent process whereby the parent either signed or asked the teacher to sign on their behalf. In situations where the parent did not fill out the consent form or communicate their consent to the teacher, a phone call was made to verify that consent had been given. The parents of 25 children refused consent in this process. Children were able to opt out at any point in time and were given a small gift of stationary regardless of their participation.

### 2.2 Sample size calculation

The results of previous clinical trials showed that around 70% of the active dentin caries became arrested with two applications after 12 months (Seifo et al., 2019). An absolute difference of 10% in the caries arrest rates between treatment groups was considered clinically significant. G‐power (version 3.1.9.2) was used to calculate the implied power given the sample size of 421 participants and the minimum effect size that would be considered to be clinically significant (0.1). The implied power was 0.959 meaning that there was less than 5% chance of failing to reject a false null hypothesis.

Children were included in the study who: (1) attended one of the four target schools, both of which had similar socioeconomic characteristics; (2) had one or more active carious lesions in primary teeth not involving the pulp, and (3) were aged less than 12‐years. Children were excluded if: (1) parents refused consent, (2) they did not assent, (3) they were outside the target age‐range, and (4) they had no teeth eligible for silver fluoride treatment. Inclusion in the study was validated by the use of calibrated examiners and then later baseline data from potential participants were validated against inclusion criteria. Overall 421 children were recruited to the study, representing 4606 primary tooth surfaces that met the criteria of: (1) ICDAS code 3 or above; and (2) not associated with a pulpally involved lesion, and (3) not expected to exfoliate soon.

### 2.3 Clinical procedures

Clinical examination was performed by visual inspection under torch light, with children in a supine position. Probing of cavitated lesions (ICDAS code 5 and 6 lesions) was performed using a ball‐ended World Health Organization CPI probe. Each child was assigned a unique identifier at baseline in order to anonymously track them throughout the trial. Data analysis was performed on a deidentified dataset only. A full‐mouth, surface level charting was performed at baseline, at the 6‐month follow‐up, and at the 12‐month follow‐up; data were collected on tooth status using the ICDAS index (Ma et al., 2020). In addition data on the color of the cavity was collected using a standardized color scale, and the center of the lesion was gently probed to gain a tactile measure of hardness across a scale of three gradations (soft, leathery, and hard). Oral hygiene status was examined by observing the presence or absence of visible plaque covering more than one‐third of the labial surface of two central incisors.

There were two separate groups of clinicians involved in the study at each time point; one group made up of four calibrated examiners and four assistants (total of six calibrated examiners across three‐time points), and a second group made up of four clinical operators and four assistants (total of 12 clinical operators across three‐time points). This meant that while most of the clinicians were active at the three‐time points, some were unable to join the activities at later time points and as such the number of examiners and clinicians who had been involved grew over the course of the trial.

Children were first examined by an examiner and then received the topical silver fluoride therapy by a clinical operator. Calibrated examiners underwent a 4‐h training session with testing at each time point to achieve an interexaminer kappa score > 0.85 (Range: 0.88–0.96) for the ICDAS index, which indicates near‐perfect agreement. Examiners were calibrated prior to each stage of the clinical trial. Intraexaminer reliability was not able to be performed due the possibility of the treatment altering the observed characteristics of the lesion and logistical constraints which limited the possibility of reexamination of the same children.

**FIGURE 1:**
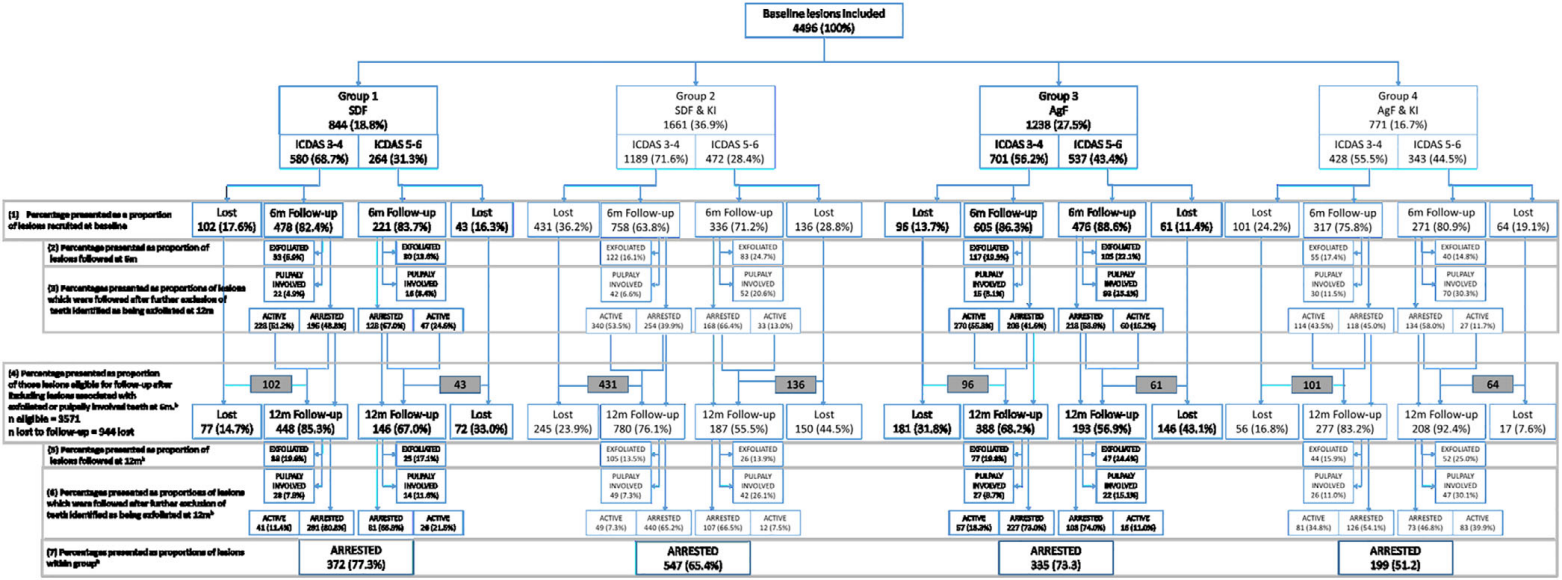
Attrition analysis and arrest outcomes by group and lesion size. ^a^Percentages are presented as proportions of certain subsections as described by shaded boxes. ^b^ *p*‐value < .001; chi‐squared test for differences in clinical outcomes by group membership

Clinical operators received 2‐h of training on how to use the open‐source data collection tool (KoBo Collect v1.25.1; KoboToolBox, MA, USA), as well as on the appropriate placement of the AgF/SDF/KI material(s) according to group membership. Silver fluoride was applied at baseline and also immediately following the 6‐month and 12‐month follow‐up examination. The clinical protocol varied according to group (Figure 1). Group 1 and Group 3 received a single step therapy with variation in the type of silver fluoride (SDF or AgF) therapy. After isolating the tooth and drying the cavity with cotton pellets, SDF or AgF was placed; moisture control was maintained for 1 min. Children in Group 2 and Group 4 received a two‐step procedure in which a silver fluoride (SDF or AgF) solution was placed on the lesion (after drying) followed by Potassium Iodide (KI). Moisture control was maintained for 1‐min after the application. Children in Group 1 and Group 2 received SDF, and children in Group 3 and Group 4 received AgF.

Children received an application of silver fluoride at three‐time points; baseline, 6 months, and following the final study examination at 12 months. After each clinical application the child was checked twice to ensure that no adverse events had occurred; once immediately after the procedure and a second time, in the classroom the following day. At each check, children were asked to report if they had experienced any discomfort and, if so, then the intraoral soft tissues were examined for signs of chemical burns. Adverse events were documented on a standardized adverse events form. The clinical procedure for isolation was modified to include placement of Vaseline on soft tissue in the anterior zone, after three adverse events were associated with migration of treatment solutions onto soft tissues during the treatment of anterior smooth‐surface lesions, causing a superficial chemical irritation.

### 2.4 Data analysis

**FIGURE 2:**
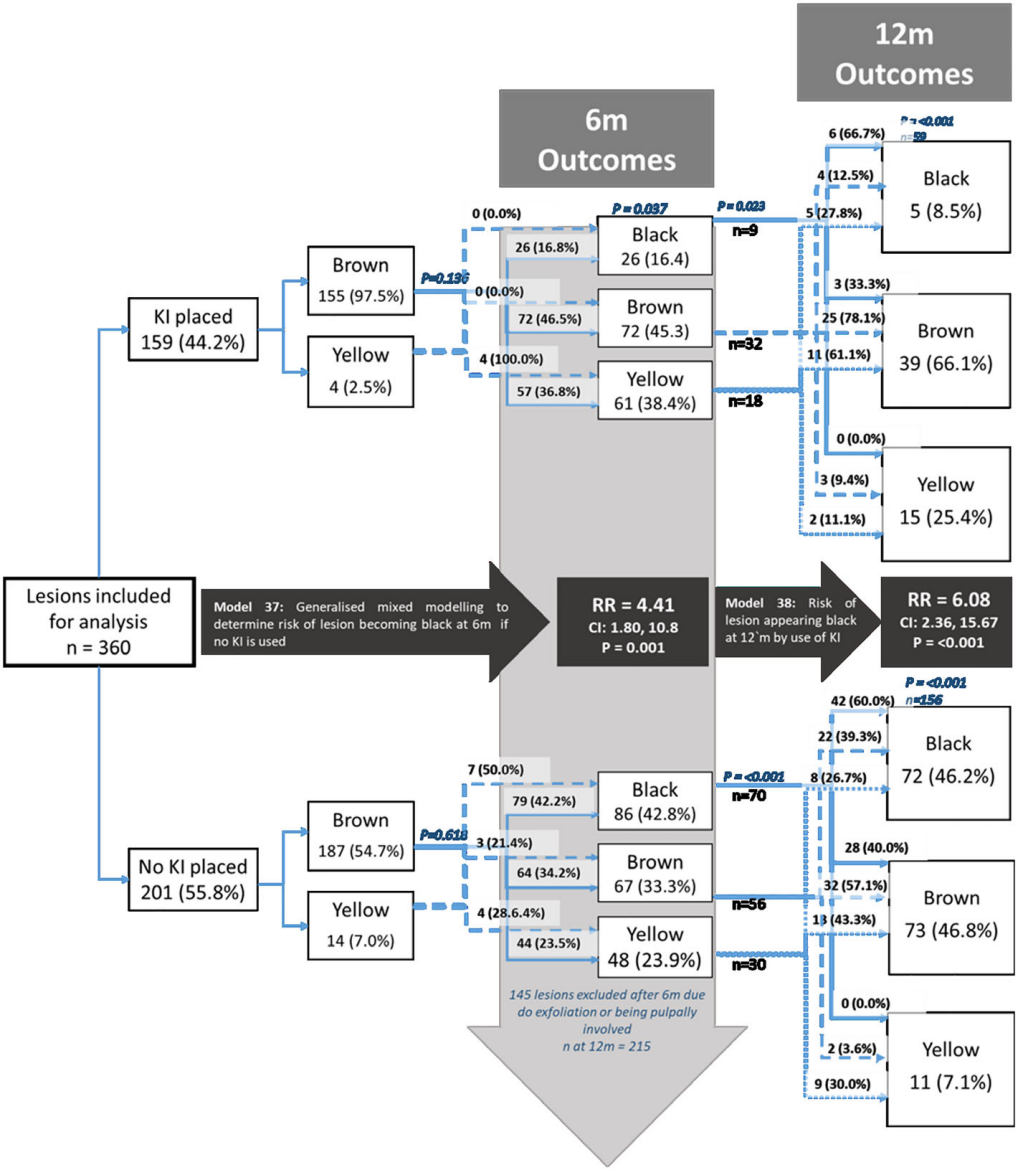
Changes in color among ICDAS 5–6 lesions at baseline, 6‐month and 12‐month follow‐up^a^. ^a^ *P*‐values presented based on chi‐squared tests for differences in proportions among groups within the same column or differences in odds of black colour developing in the case of model 37 and model 38

The primary outcome of interest was the arrest of the carious lesion at surface level which was based on (a) a change in size of a lesion using the ICDAS index, and (b) the hardness of a lesion. These criteria were applied differently depending on the clinical presentation of the lesion at baseline. Those lesions which were ICDAS code 3 or code 4 at baseline were judged to have arrested if at the 6‐month follow‐up they had not transitioned to a more severe ICDAS code. In the case of ICDAS code 6 lesions, the lesion had to be “hard” rather than “leathery” or “soft” in order to qualify as arrested. For a lesion that was ICDAS 5 at baseline, it was classified as being arrested if it was hard or if it was stable in size. Arrest at the 6‐month time point was considered against baseline status. At 12‐month arrest was compared against the 6‐month status and for those lesions which were observed at 12 months but not at 6 months then lesion status was compared with the baseline status. Teeth with lesions that were stable in size but which showed signs of sepsis according to the PUFA index were judged as being “caries active.” The outcome of color did not form part of the arrest criteria in this study since the addition of KI in two groups is known to reduce the discoloration caused by the silver fluoride solution (Figure 2).

There were two secondary outcomes of interest; whether or not a lesion became a “black” color and whether or not a tooth became nonvital. The darkening of the lesion was assessed according to a color scale incorporating categories representing “yellow”, “brown,” and “black”. For the analysis of color change, only lesions where dentine was visible were included (ICDAS code 5 and code 6). Lesions which were judged as black at baseline or were not followed at 6 months were excluded from the color‐change analysis. Lesion scores were aggregated at tooth level. Teeth were assigned a PUFA index score (indicating pulpal involvement) if the caries had progressed to a state at which the pulp chamber was visible, ulceration of the adjacent soft tissue was seen, or there was a fistula or abscess. Teeth (and those lesions associated with a particular tooth) that were deemed as being pulpally involved at 6 months were not included in the 12‐month follow‐up analysis.

Survey data were collected on the Kobo Collect app and clinical data were collected on paper and then entered into an Excel spreadsheet. Data were transferred and analyzed using SPSS Version 23 (IBM Corp, NY, USA). Differences in proportions among groups were compared using a chi‐square test and differences in means between groups were compared using the Kruskal–Wallis test for nonparametric data.

Two‐level modeling was used to allow for the clustering effect of multiple lesions that might occur within one individual. The first level of the models was used to cluster control for individual‐level variables and the second level included lesion level effects. The covariates included were sociodemographic characteristics (sex and age), tooth type (molar or incisor), lesion position (which surface of the tooth), baseline caries experience (divided into tertiles), and plaque scores, at each follow‐up. The predictor variables were group membership, type of silver fluoride therapy used, and whether or not KI was used. There were 14 models built to examine outcomes of caries arrest, pulpal involvement, and transition to a black color and subcategorized by follow‐up time period and lesion size, where the ICDAS codes 3 and 4 lesions were considered separately to larger lesions.

## 3 RESULTS

A total of 901 children were screened and of that number, 25 refused consent, 74 were not eligible due to age, 124 were not eligible due to clinical presentation, and 257 were excluded due to being absent at the time of clinical treatment or unable to participate in the clinical examination due to time constraints. There were 421 children recruited into the trial with a mean age of 7.6 (SD1.9) years; 79.1% and 75.4% of children were followed at 6 months and 12 months, respectively (Table 1). Those in group 4 had a statistically significant (*p*‐value < .05; chi‐squared test) lower age and there was a significant difference in follow‐up by age‐group at both time points whereby those in the youngest (<6 years) and oldest (>11 years) age‐groups had a lower chance of being followed. There were also some significant differences in follow‐up whereby groups 3 and 4 had higher follow‐up at 6 month and group 3 had a lower follow‐up at 12 month.

**TABLE 1:** Attrition analysis of individuals by group membership and sociodemographic characteristics

	Baseline *N* (column %)	Followed 6 months *N* (row %)	Followed 12 months *N* (row %)	6 months and 12 months *N* (row %)
Group membership				
Group 1—SDF	83 (19.7)	65 (78.3)	66 (79.5)	56 (67.5)^a^
Group 2—SDF and KI	148 (35.2)	108 (73.0)	110 (74.3)	79 (53.4)
Group 3—AgF	122 (29.0)	105 (86.1)	85 (69.7)	79 (64.8)
Group 4—AgF and KI	68 (16.2)	55 (80.9)	57 (83.8)	47 (69.1)
Sex				
Male	224 (53.2)	178 (79.5)	173 (77.2)	146 (65.2)
Female	197 (46.8)	155 (78.7)	145 (73.6)	115 (58.4)
Age‐group^a^				
3‐years	13 (3.1)	9 (69.2)^a^	8 (61.5)^a^	7 (2.7)^a^
4‐years	5 (1.2)	2 (40.0)	3 (60.0)	1 (20.0)
5‐years	39 (9.3)	17 (43.6)	32 (82.1)	15 (38.5)
6‐years	61 (14.5)	46 (75.4)	46 (75.4)	36 (59.0)
7‐years	82 (19.5)	69 (84.1)	62 (75.6)	54 (65.9)
8‐years	94 (22.3)	85 (90.4)	82 (87.2)	76 (80.9)
9‐years	45 (10.7)	37 (82.2)	32 (71.1)	27 (60.0)
10‐years	49 (11.6)	42 (85.7)	33 (67.3)	30 (61.2)
11‐years	33 (7.8)	26 (78.8)	20 (60.6)	15 (45.5)
Total	421 (100.0)	333 (79.1)	318 (75.5)	261 (62.0)

^a^ *p* = .005; *χ* ^2^ test for differences among groups within the same column.

At baseline, there were three adverse events due to migration of treatment solutions onto soft tissues causing transient gingival irritation. There were no adverse events at the 6‐month and 12‐month follow‐up.

There were significant differences in lesion appearance at baseline by group membership (Table 2). Lesions in groups 1 and 2 were more likely to be on incisor teeth and there was a higher proportion of ICDAS code 5 and code 6 lesions in groups 3 and 4. There were also differences in the proportion of Eligible lesions present among younger individuals by group. Those in group 2 and group 4 had a higher proportion of lesions included in the study compared with older participants.

**TABLE 2:** Baseline clinical characteristics of lesions by group membership

	Group 1 *n* (%)	Group 2 *n* (%)	Group 3 *n* (%)	Group 4 *n* (%)	Overall *N* (%)
Tooth type^a^					
Molar lesion	433 (51.3)	867 (52.2)	725 (58.6)	438 (58.2)	2463 (54.8)
Incisor lesion	411 (48.7)	794 (47.8)	513 (41.4)	315 (41.8)	2033 (45.2)
Surface type					
Occlusal	120 (13.9)	232 (13.5)	173 (13.6)	114 (17.8)	639 (13.8)
Proximal	523 (60.7)	1059 (61.5)	812 (63.7)	450 (58.4)	2844 (61.4)
Smooth surface	219 (25.4)	431 (25.0)	289 (22.7)	207 (26.8)	1146 (24.8)
Baseline ICDAS code^a^					
Code 3	347 (40.3)	868 (50.4)	382 (30.0)	279 (36.2)	1876 (40.5)
Code 4	242 (28.1)	353 (20.5)	334 (26.2)	149 (13.8)	1078 (23.3)
Code 5	154 (17.9)	254 (14.8)	238 (18.7)	109 (14.1)	755 (16.3)
Code 6	119 (13.8)	247 (14.3)	320 (25.1)	234 (30.4)	920 (19.9)
Color at baseline^b^					
Yelow	20 (7.3)	18 (3.6)	23 (4.1)	14 (4.1)	75 (4.5)
Brown	215 (78.8)	416 (83.0)	447 (80.1)	286 (83.4)	1364 (81.4)
Black	38 (13.9)	67 (13.4)	88 (15.8)	43 (12.5)	236 (14.1)
Hardness at BL^b^					
Soft	85 (31.1)	121 (24.2)	259 (46.4)	166 (48.4)	631 (37.7)
Leathery	145 (53.1)	263 (52.5)	108 (19.4)	82 (23.9)	598 (35.7)
Hard	43 (15.8)	117 (23.4)	191 (34.2)	95 (27.7)	446 (26.6)
Sex^a^					
Male	400 (46.4)	861 (50.0)	695 (54.6)	409 (53.0)	2365 (51.1)
Female	462 (53.6)	861 (50.0)	579 (45.4)	362 (47.0)	2264 (48.9)
Age‐group^a^					
3‐years	42 (4.9)	0 (0.0)	107 (8.4)	0 (0.0)	149 (3.2)
4‐years	39 (42.9)	52 (3.0)	0 (0.0)	0 (0.0)	91 (2.0)
5‐years	141 (16.4)	356 (20.7)	0 (0.0)	138 (17.9)	635 (13.7)
6‐years	171 (19.8)	298 (17.3)	360 (28.3)	198 (25.7)	1027 (22.2)
7‐years	140 (16.2)	401 (23.3)	320 (25.1)	187 (24.3)	1048 (22.6)
8‐years	171 (19.8)	239 (13.9)	258 (20.3)	214 (27.8)	882 (19.1)
9‐years	35 (4.1)	180 (10.5)	145 (39.6)	6 (0.8)	336 (7.9)
10‐years	89 (10.3)	148 (8.6)	40 (3.1)	4 (0.5)	281 (6.1)
11‐years	34 (3.9)	48 (2.8)	44 (3.5)	24 (3.1)	150 (3.2)
Total (row %)	844 (18.8)	1611 (36.9)	1238 (27.5)	753 (16.7)	4496 (100.0)

^a^ Brackets contain column percentages unless otherwise indicated. There were statistically significant differences in clinical characteristics by group membership across all descriptors in the table; Group 1 = SDF; Group 2 = SDF&KI; Group 3 = AgF; Group 4 = AgF&KI.

^b^ Among lesions ICDAS > 5 at baseline *n* = 1675.

Figure 1 presents data on retention of the 4496 lesions and arrest outcomes across the course of the trial. There were significant differences in loss to follow‐up by group membership (Tables 1 and 2); those in group 3 had a greater loss to follow‐up at 12 month. Bivariate analysis suggests that differences in arrest rates were more pronounced at 12 months and among smaller lesions, whereby those lesions present in group 2 and group 4 (where KI was applied) had a lower proportion of arrested lesions than other groups.

Two‐level generalized mixed models were used to examine caries arrest at each time point by lesion size, group membership, SDF type, and use of KI. The use of KI (when modeled as a predictor or when considered separately in group 2 and group 4) was associated with approximately four times lower odds of arrest at 12 months (Table 3).

**TABLE 3:** Generalized mixed model for caries arrest rate among at 6 months and 12 months by lesion size, group membership, SDF type, and use of KI^a^

	All lesions (ICDAS 3–6)					
	Arrest of caries at 6 months			Arrest of caries at 12 months		
	Odds ratio	95% CI	*p*‐value	Odds ratio	95% CI	*p*‐value
Group						
Group 1^b^	Model 1			Model 2		
Group 2	0.77	0.59, 1.01	.062	0.21	0.14, 0.30	<.001
Group 3	1.00	0.75, 1.33	.999	0.64	0.40, 1.02	.063
Group 4	0.77	0.56, 1.07	.126	0.18	0.11, 0.29	<.001
SDF type						
SDF^b^	Model 3			Model 4		
AgF	1.07	0.87, 1.31	.0539	1.16	0.88, 2.26	.149
Use of KI						
No KI^b^	Model 5			Model 6		
Has KI	0.77	0.63, 0.94	.011	0.25	0.19, 0.34	<.001

^a^ Two‐level modeling was used, the first level of the models was used to cluster control for individual‐level variables and the second level included lesion level effects. The individual‐level variables were based on the individual unique identifier, age, and sex. The covariates included were tooth type (molar or incisor), lesion surface, baseline caries experience, and oral hygiene.

^b^ Reference category.

Table 4 presents data on the proportion of teeth which became pulpally involved over the course of observation. Between baseline and 6‐month follow‐up 167 teeth in 109 children became pulpally involved (10.3%). Between 6‐ and 12‐month follow‐up a further 146 teeth (11.7%) in 97 children became pulpally involved. Those subgroups where KI had been used had a higher proportion of teeth which became pulpally involved. That effect was particularly visible at the 12‐month time point where 31.8% of teeth that were treated with KI became pulpally involved compared with 13.3% of teeth where KI was not used. Those teeth which had ICDAS code 5 or code 6 lesions and were treated with KI were the most likely to develop pulpal involvement.

**TABLE 4:** Number and prevalence of new pulpally involved teeth by baseline lesion size, group membership, SDF type, and use of KI at 6 months and 12 months

	6‐month outcomes: *n* (row %)				12‐month outcomes: *n* (row %)			
	*n*	ICDAS 3–4	ICDAS 5–6	All teeth	*n*	ICDAS 3–4	ICDAS 5–6	All teeth^a^
Group								
Group 1	353	12 (4.3)^b^	9 (12.2)	21 (5.9)	284	22 (9.4)	6 (12.0)^b^	28 (9.9)^b^
Group 2	497	24 (6.0)	23 (24.5)	47 (9.5)	511	35 (7.9)	21 (30.0)	56 (11.0)
Group 3	508	9 (2.6)	45 (26.8)	54 (10.6)	273	16 (7.9)	10 (14.3)	26 (9.5)
Group 4	262	18 (10.7)	27 (29.0)	45 (17.2)	184	16 (12.8)	20 (33.9)	36 (19.6)
SDF type								
SDF	850	36 (5.3)	32 (19.0)^b^	68 (8.0)^b^	457	32 (9.8)	30 (23.3)	62 (13.6)
AgF	770	27 (5.3)	72 (27.6)	99 (12.9)	795	57 (8.4)	27 (22.5)	84 (10.6)
Use of KI								
No KI	861	21 (3.4)^b^	54 (22.3)	75 (8.7)	557	38 (8.7)	16 (13.3)^b^	54 (9.7)^b^
Has KI	759	42 (7.3)	50 (26.7)	92 (12.1)	695	51 (9.0)	41 (31.8)	92 (13.2)
Total								
Vital	–	1128 (94.7)	325 (75.8)	1453 (89.7)	–	914 (91.1)	192 (77.1)	1106 (88.3)
Nonvital	–	63 (5.3)	104 (24.2)	167 (10.3)	–	89 (8.9)	57 (22.9)	146 (11.7)

^a^ Teeth identified as being pulpally involved at 6 month are excluded from the 12‐month analysis.

^b^ *p* ≤ .005; Chi‐squared tests for difference in proportions among groups within the same column.

The multivariate modeling further confirmed these findings whereby those teeth which had KI placed had around twice the odds of becoming pulpally involved (Table 5). Teeth in Group 4 had the highest odds of developing new pulpal lesions when compared with group 1; OR 2.54 and 3.02 at 6 months and 12 months, respectively.

**TABLE 5:** Generalized mixed model for caries progression of a lesion to pulpally involved status by lesion size, group membership, SDF type, and use of KI at 6 months and 12 months

	ICDAS 3–6					
	Presence of new pulpally involved lesions 6 month			Presence of new pulpally involved lesions 12 month		
	Odds ratio	95% CI	*p*‐value	Odds ratio	95% CI	*p*‐value
Group^a^						
Group 1	Model 7			Model 10		
Group 2	1.38	0.69, 2.76	.358	1.23	0.62, 2.42	.588
Group 3	1.30	0.66, 2.56	.456	0.534	0.23, 1.25	.150
Group 4	2.54	1.2, 5.13	.009	3.02	1.36, 6.73	.007
SDF type						
SDF^a^	Model 8			Model 11		
AgF	1.36	0.88, 2.12	.168	0.98	0.59, 1.63	.933
Use of KI						
No KI^a^	Model 9			Model 12		
Has KI	1.51	0.99, 2.30	.057	2.21	1.33, 3.68	.002

^a^ Group 1 = SDF; Group 2 = SDF&KI; Group 3 = AgF; Group 4 = AgF&KI.

Lesions which were ICDAS code 5 and code 6, which were not black in color at baseline, and were followed at the two follow‐up time points, were included for analysis of color change over time (*n* = 360). Lesions which were treated with KI had four times lower odds and six times lower odds of becoming black at the 6‐month and 12‐month follow‐up, respectively.

## 4 DISCUSSION

This four‐armed, parallel‐design randomized controlled trial examined differences in lesion arrest, lesion color, and loss of tooth vitality (development of pulpal involvement) by group membership, type of silver fluoride solution, and addition of KI. It found that while the KI has a sixfold chance of avoiding black discoloration compared with silver fluoride therapies on their own, it was also associated with a lower arrest rate and higher odds that a tooth might become pulpally involved. Both silver fluoride solutions performed with similar results across the three outcomes examined. These findings will inform HKC program delivery in that, the use of KI will be limited to anterior tooth lesions and both AgF and SDF might be used interchangeably with confidence. Before further examination of the findings of the study it is appropriate to first consider the strengths and limitations of the study.

Performing clinical trials in challenging environments such as those that exist in Cambodia can yield important information, however, balancing ethical considerations can make it difficult to maintain ideal experimental conditions. The four schools selected were all due to enter the HKC Program in 2020. They were randomly assigned, at school level, to one of the four treatment groups. There were however differences in caries experience among the children in each school and the school with the most severe caries experience was the school (group 4) with the highest proportional loss to follow‐up. There were 558 lesions in group 4 observed at the 12‐month follow‐up which exceeds the required sample size of 363 lesions; however, it is acknowledged that this loss to follow‐up could have contributed to some bias in the comparison of clinical outcomes among groups, whereby outcomes in other groups may have appeared to be more favorable. The individual children within those schools were not randomly assigned into the treatment groups, the caries experience was unequal, and all children at the school were given the same treatment; this means that the examiners were not blinded. This could lead to bias due to the fact that some examiners may have been tempted to score lesions differently knowing the type of treatment that was provided.

The weaknesses in sample selection design have been partly mitigated by appropriate statistical methods to control for clustering and differences among groups. The strengths of the study include that an adequate sample size was selected and that follow‐up rates were high, which meant that this study has sufficient statistical power to examine the research questions that were posed. Another strength of the study design was the use of ICDAS codes to quantify the stability of the size of the lesion as a criteria for arrest. Some previous studies have determined arrest based only on hardness and color, which are difficult to calibrate. In the present study, the calibrated examiners could provide an accurate measure of lesion size.

One of the more unique aspects of this study was the use of the PUFA index as an outcome measure and this was thought to be necessary due to the fact that these children have a severe burden of dental caries and very little access to dental care outside the HKC program. Therefore, the therapeutic aim was not just to arrest carious lesions but also to avoid a situation whereby a child has an episode of severe pain or infection associated with a pulpally involved tooth. The results from the present study suggest that further investigation is needed to better understand which teeth might be more likely to fail after silver fluoride therapy. Other data from an investigation involving delayed application of SDF among older children (mean age > 8 years) suggested that failing to place SDF leads to a 1 in 5 incidence of pulpally involved lesions after 12 months, rather than a 1 in 10 incidence as reported in the present study (Ma et al., 2020). For ethical reasons, this study did not have a “no treatment” control group where the caries arrest, caries progression, and pulpal involvement could be compared with the intervention groups. And so it is not known if this rate of one in 10 carious teeth becoming pulpally involved is an improvement on what might have otherwise occurred with no intervention. Although children may have experienced better overall outcomes than had they not had the silver fluoride treatment, it appears that for around 100 of the 421 children this was not sufficient to prevent the loss of vitality of at least one treated tooth. If it were possible for those lesions which are not likely to arrest with silver fluoride treatment to be identified during the triage process, then perhaps allocation of treatment resources to restore these teeth within the HKC program could be considered.

Lesion size by ICDAS coding was also important for both the analysis of lesion progression and for the formulation of clinical advice. Lesion size is a key consideration in case selection and placement of silver fluoride and/or KI. Lesion size also influences the cleansability and biomechanics of a lesion. For example, an ICDAS code 4 lesion is defined as “an underlying dark shadow from dentine” (dos Santos et al., 2015) and such lesions may behave differently to code 5 and 6 lesions from a biomechanical and chemo‐therapeutic point of view. Although the demineralized enamel on the surface of an ICDAS 4 lesion may benefit from application of silver therapies, the body of the lesions are not accessible to microbrush application. Hence, when groups of children from different schools have differences in the proportions of teeth with different lesion sizes, this can confound the arrest rate outcomes; this phenomenon was apparent in the 6‐month results (Turton, Horn et al., 2020). Although the inclusion of less severe lesions is not customary in SDF studies, it was appropriate in this case, where the data inform program delivery to underserved communities. In the HKC program, the children would not be likely to receive restorative care for those lesions, and so it was important to confirm that 70%–80% of those ICDAS Code 3–4 lesions would remain stable over a 12‐month period of time.

Two key findings of the present study were that the arrest rates varied by lesion size and by the placement of KI. In previous reporting of this clinical trial at 6 months, there were some statistically significant results which included that those in group 4 (AgF and KI) had higher arrest rates. However, some caution was advised in that paper due to possible confounding in that those in group 4 had a higher proportion of larger lesions. In other words, where a group had a higher proportion of ICDAS code 3 and code 4 lesions then the biomechanical properties of such lesions would lead to breakdown of undermined enamel over time. As such, these lesions would have a higher chance of being reported as “active”, and at group level, attributing differences in arrest to the therapeutic effect of the solutions might be confounded (Turton, Horn et al., 2020). Hence at the 12‐month time point failure to arrest was defined as any further breakdown between the 6‐month time point and the 12‐month time point. In that way, the confounding that occurred because of initial breakdown would be less likely to create bias as lesions stabilized. The 12‐month results suggest that those lesions treated with KI had a lower chance of arrest after taking into account the effect of other covariates.

The mechanism by which the KI reduces arrest rate was not explored in this study and further investigations could examine differences in the histological and biomechanical properties of dentine lesions treated with KI. When KI is placed on a carious lesion that has been treated with AgF it reacts with the silver creating silver iodide (AgI). In that way, excess silver ions which might otherwise contribute to staining are removed. Other authors have suggested that the removal of silver ions might reduce the “anti caries” effect (Zhao et al., 2017), and the present study supports this hypothesis. It could also be that the application of KI might prevent the “micro‐wire formation” which has been documented when silver fluoride therapies are applied (Seto et al., 2017). An absence or reduction of microwire formation could lead to a less favorable outcome from a biomechanical or caries control standpoint.

Although the caries arrest rate might be lower when KI is used, there are still benefits to be gained by using it, such as the six times lower odds of the lesion becoming black. The improved lesion appearance has been reported in other in vitro studies although the quality of the evidence is limited (Roberts et al., 2020). In addition, there are reports that KI might have benefits for desensitization of dentine through promoting the occlusion of dentinal tubules (Craig et al., 2012). Presently there are no other published investigations examining the reduction in staining of AgF‐treated carious lesions on primary teeth following use of KI. And studies on the perceptions of children and their parents on the improved appearance following KI treatment have yet to be conducted. Clinicians using silver fluoride solutions to treat primary tooth caries have a choice of whether to include the KI step or not. They need to weigh up the potential benefits (lesion appearance and reduction in sensitivity), against a lower chance of lesion arrest. Clinicians may choose to limit the use of KI to lesions which have both high aesthetic importance (e.g., anterior teeth) and lesions which have a cleansable, morphology that favors caries arrest. For example, an ICDAS code 5, smooth surface lesion on an anterior tooth.

## 5 CONCLUSIONS

This study demonstrated that both AgF and SDF can effectively arrest carious lesions on primary teeth as consistent with other reported literature. The use of KI reduced the staining, however, it also reduced the chances of caries arrest. A higher proportion of lesions progressed to involve the pulp over a 12‐month period in those teeth where KI was used. This study suggests that use of KI would be best limited to lesions on anterior teeth where lesion morphology is more favorable, the chance of arrest is higher, and aesthetics are more important. Further investigation is needed to explore the reasons why some lesions continue to progress to pulpal involvement following AgF/SDF application, in order to avoid children in the HKC program developing pain and infection.

## CONFLICT OF INTEREST

The investigators declare that there is a conflict of interest in the conduct of the study because the lead investigator was commissioned as a consultant by the manufacturer of the products being tested (SDI Limited). This conflict is partly mitigated by the fact that the HKC project has been using SDF therapy since 2014 and the payment was consistent with local expectations of salary for conducting this type of work in a Cambodian environment through local agencies. Deidentified data remain the property of the investigators' institution and this project could be considered a partnership between the dental company and the existing HKC project to improve the quality of care for participating children.

## AUTHOR CONTRIBUTIONS

Bathsheba Turton contributed to study design, field work, data analysis, and drafting of the article. Rithvitou Horn contributed to field work and interpretation of results in the article. Callum Durward contributed to study design, interpretation of results, and editing of the article.

## Data Availability

Data are available for secondary analysis upon reasonable request.

## References

[cre2367-bib-0001] Arrow, P. (2015). Restorative outcomes of a minimally invasive restorative approach based on atraumatic restorative treatment to manage early childhood caries: A randomised controlled trial. Caries Research, 50, 1–8. 26667118 10.1159/000442093

[cre2367-bib-0002] Chu, C. H. , & Lo, E. C. (2008). Promoting caries arrest in children with silver diamine fluoride: A review. Oral Health & Preventive Dentistry, 6, 315–321. 19178097

[cre2367-bib-0003] Craig, G. G. , Knight, M. , & McIntyre, J. M. (2012). Clinical evaluation of diamine silver fluoride/potassium iodide as a dentine desensitizing agent: A pilot study. Australian Dental Journal, 57, 308–311. 22924353 10.1111/j.1834-7819.2012.01700.x

[cre2367-bib-0004] Crystal, Y. O. , Janal, M. N. , Hamilton, D. S. , & Niederman, R. (2017). Parental perceptions and acceptance of silver diamine fluoride staining. Journal of the American Dental Association, 148, 510–518. 28457477 10.1016/j.adaj.2017.03.013 PMC6771934

[cre2367-bib-0005] dos Santos, S. E. S. , Bezerra, A. C. B. , de Amorim, R. F. S. G. , & Leme, T. C. P. (2015). Caries diagnosis in the mixed dentition using ICDAS II. Pesqui Bras Em Odontopediatria E Clínica Integrada, 18, 13–21.

[cre2367-bib-0006] Duangthip, D. , Jiang, D. , Chu, C. H. , & Lo, E. C. (2015). Non‐surgical treatment of dentin caries in preschool children? Systematic review. BMC Oral Health, 15, 44. 25888484 10.1186/s12903-015-0033-7 PMC4403709

[cre2367-bib-0007] Fung, M. , Duangthip, D. , Wong, M. , Lo, E. , & Chu, C. (2016). Arresting dentine caries with different concentration and periodicity of silver diamine fluoride. JDR Clinical & Translational Research, 1, 143–152. 28989974 10.1177/2380084416649150 PMC5615850

[cre2367-bib-0008] Knight, G. , Mclntyre, J. , & Craig, G. (2006). Ion uptake into demineralized dentine from glass ionomer cement following pretreatment with silver fluoride and potassium iodide. Australian Dental Journal, 51, 237–241. 17037890 10.1111/j.1834-7819.2006.tb00435.x

[cre2367-bib-0009] Ma, C., Bun, K., Sok, P., Turton, B., & Tak, R . (2020). *Management of advanced carious lesions in primary teeth for children in late mixed dentition; An observational study from the Healthy Kids Cambodia project*. (Thesis for the fulfilment of Dental Doctorate Degree). University of Puthisastra, Cambodia.

[cre2367-bib-0010] Roberts, A. , Bradley, J. , Merkley, S. , Pachal, T. , Gopal, J. V. , & Sharma, D. (2020). Does potassium iodide application following silver diamine fluoride reduce staining of tooth? A systematic review. Australian Dental Journal, 65, 109–117. 31900927 10.1111/adj.12743

[cre2367-bib-0011] Seifo, N. , Cassie, H. , Radford, J. , & Innes, N. (2019). Silver diamine fluoride for managing carious lesions: An umbrella review. BMC Oral Health, 19, 145. 31299955 10.1186/s12903-019-0830-5 PMC6626340

[cre2367-bib-0012] Seto, J. , Horst, J. , Parkinson, D. , Frachella, J. , & DeRisi, J. (2017). Silver microwires from treating tooth decay with silver diamine fluoride. BioRxiv, 1, 152199.

[cre2367-bib-0013] Slayton, R. L. , Urquhart, O. , Araujo, M. W. B. , Fontana, M. , Guzmán‐Armstrong, S. , Nascimento, M. M. , … Young, D. A. (2018). Evidence‐based clinical practice guideline on nonrestorative treatments for carious lesions: A report from the American Dental Association. Journal of the American Dental Association, 149, 837–849. 30261951 10.1016/j.adaj.2018.07.002

[cre2367-bib-0014] Tirupathi, S. , Nirmala, S. V. S. G. , Rajasekhar, S. , & Nuvvula, S. (2019). Comparative cariostatic efficacy of a novel nano‐silver fluoride varnish with 38% silver diamine fluoride varnish a double‐blind randomized clinical trial. Journal of Clinical and Experimental Dentistry, 11, e105–e112. 30805113 10.4317/jced.54995 PMC6383905

[cre2367-bib-0015] Turton, B. , Horn, R. , & Durward, C. (2020). Caries arrest and lesion appearance using two different silver fluoride therapies with and without potassium iodide: 6‐month results. Heliyon, 6, e042287. 10.1016/j.heliyon.2020.e04287 PMC7369606 32715116

[cre2367-bib-0016] Turton, B. , Patel, J. , Hill, R. , Sieng, C. , & Durward, C. (2020). Healthy Kids Cambodia: A novel approach to triage for dental care in a population with extreme caries experience. Community Dentistry and Oral Epidemiology, 48, 56–62. 31734941 10.1111/cdoe.12503

[cre2367-bib-0017] Urquhart, O. , Tampi, M. P. , Pilcher, L. , Slayton, R. L. , Araujo, M. W. B. , Fontana, M. , … Carrasco‐Labra, A. (2019). Nonrestorative treatments for caries: Systematic review and network meta‐analysis. Journal of Dental Research, 98(2019), 14–26. 30290130 10.1177/0022034518800014 PMC6304695

[cre2367-bib-0018] Yee, R. , Holmgren, C. , Mulder, J. , Lama, D. , Walker, D. , & van Palenstein Helderman, W. (2009). Efficacy of silver diamine fluoride for arresting caries treatment. Journal of Dental Research, 88, 644–647. 19641152 10.1177/0022034509338671

[cre2367-bib-0019] Zhao, I. S. , Mei, M. L. , Burrow, M. F. , Lo, E. C. , & Chu, C. H. (2017). Effect of silver diamine fluoride and potassium iodide treatment on secondary caries prevention and tooth discolouration in cervical glass ionomer cement restoration. International Journal of Molecular Sciences, 18, 340. 10.3390/ijms18020340 PMC5343875 28178188

